# Exosomal lncRNAs in Cerebrovascular Diseases: Biomarkers, Pathological Mechanisms, and Therapeutic Potential

**DOI:** 10.3390/ncrna12050032

**Published:** 2026-08-24

**Authors:** Haiyu Su, Daiju Tao, Jia Teng, Li Zhang, Ying Shen, Renhua Yang, Jiarui Yang, Rongji Sun, Zhiqiang Shen, Peng Chen, Bo He

**Affiliations:** 1 Yunnan Key Laboratory of Pharmacology for Natural Products, School of Pharmaceutical Science, Kunming Medical University, Kunming 650500, China; 18987255202@163.com (H.S.); 18087877808@163.com (D.T.); zhangli69@kmmu.edu.cn (L.Z.); 15198755271@163.com (Y.S.); yangrenhua@kmmu.edu.cn (R.Y.); 13888733168@163.com (J.Y.); sunrongjiscu@163.com (R.S.);; 2 Department of Pharmacology, Haiyuan College, Kunming 650106, China; 13987103770@163.com; 3 Yunnan Institute for Drug Abuse, Kunming 650228, China

**Keywords:** exosomal lncRNAs, cerebrovascular diseases, ischemic stroke, atherosclerosis, biomarkers

## Abstract

**Background:** Cerebrovascular diseases have complex pathogenesis and pose a serious threat to human health; thus, novel diagnostic and therapeutic strategies are needed. Small extracellular vesicles (sEVs), commonly referred to as exosomes, are 30–150 nm lipid-bilayer vesicles that shield long noncoding RNAs (lncRNAs) from degradation. Because exosomal lncRNAs are more stable than free lncRNAs in blood and cerebrospinal fluid and can cross the blood–brain barrier, they are promising as biomarkers and therapeutic vectors. This review summarizes the roles and mechanisms of exosomal lncRNAs in cerebrovascular diseases. **Methods:** This narrative review is based on the experimental literature and focuses on the biological functions and regulatory mechanisms of exosomal lncRNAs in cerebrovascular disorders. **Results:** As competing endogenous RNAs (ceRNAs), they sequester microRNAs (miRNAs), thereby derepressing downstream target-gene expression and modulating neuronal injury (oxidative stress, apoptosis, neuroinflammation) through the nuclear factor kappa-B (NF-κB) and phosphoinositide 3-kinase/protein kinase B (PI3K/Akt) pathways. They show altered profiles in acute stroke (ischemic/hemorrhagic) correlated with neurological deficits, and are relevant to the early diagnosis of chronic diseases (atherosclerosis, aneurysm) and vascular dementia. **Conclusions:** Exosomal lncRNAs demonstrate promising translational potential in preclinical studies because they combine exosome delivery capabilities with lncRNA regulatory functions, although clinical validation remains limited.

## 1. Introduction

Cerebrovascular diseases, which are major illnesses that threaten human health, have high incidence and mortality rates, and have become serious global public health challenges [1]. Cerebrovascular diseases include acute conditions, such as ischemic stroke and cerebral hemorrhage, and chronic conditions, such as vascular dementia, all of which seriously affect patients’ quality of life and impose a heavy socioeconomic burden. Although traditional treatments, such as thrombolysis, endovascular intervention and surgery, have achieved some success, limitations remain in terms of neurological function recovery, prevention of recurrence, and improvement of long-term prognosis [2]. Therefore, in-depth exploration of the molecular mechanisms of cerebrovascular diseases and the development of new diagnostic and therapeutic strategies are of significant clinical importance.

Exosomes, which are extracellular vesicles (30–150 nm), can be released from various cell types, such as vascular endothelial cells, macrophages, and glial cells [3]. Owing to their excellent biocompatibility, outstanding ability to cross the blood–brain barrier, and stability in vivo, exosomes not only are promising biomarkers for disease diagnosis and prognosis evaluation, but also serve as ideal delivery vehicles for targeted therapy [4]. Long noncoding RNAs (lncRNAs), important functional molecules packaged by exosomes, participate in the pathophysiological activities of cerebrovascular diseases through the regulation of functions such as angiogenesis, endothelial functions, smooth muscle cell phenotypes and platelet function [5]. As a critical cargo of exosomes, exosomal lncRNAs play important roles in diseases, but their molecular regulatory network has not yet been systematically characterized.

Many studies have been conducted over the past few years on noncoding RNAs (ncRNAs) in exosomes associated with neurologic diseases. Wang et al. [6] systematically discussed the diagnostic and therapeutic potential of exosomal ncRNAs in various central nervous system disorders, but their discussion on cerebrovascular diseases was limited. Wang et al. [7] focused on the application of exosomal lncRNAs and circRNAs in ischemic stroke but did not cover hemorrhagic stroke or chronic cerebrovascular lesions. Recently, Li et al. [8] provided a comprehensive overview of how miRNAs found in exosomes have protective effects on the brain; however, little information has been presented on lncRNAs. They emphasized the neuroprotective effects of exosomal miRNAs, but their discussion on lncRNAs was less comprehensive. On the basis of the limitations of the above studies, this article provides a systematic review of the research progress on exosomal lncRNAs across the full spectrum of cerebrovascular diseases, covering acute lesions (ischemic and hemorrhagic stroke) and chronic lesions (atherosclerosis, cerebral aneurysms, and vascular dementia). This review focuses on the mechanisms by which exosomal lncRNAs modulate oxidative stress, apoptosis, and neuroinflammation, with particular attention given to cell-of-origin-dependent functional differences. Additionally, we discuss how to take advantage of ncRNAs in exosomes for clinical applications with an emphasis on developing a more accurate way of diagnosing and managing cerebrovascular diseases.

**Figure 1:**
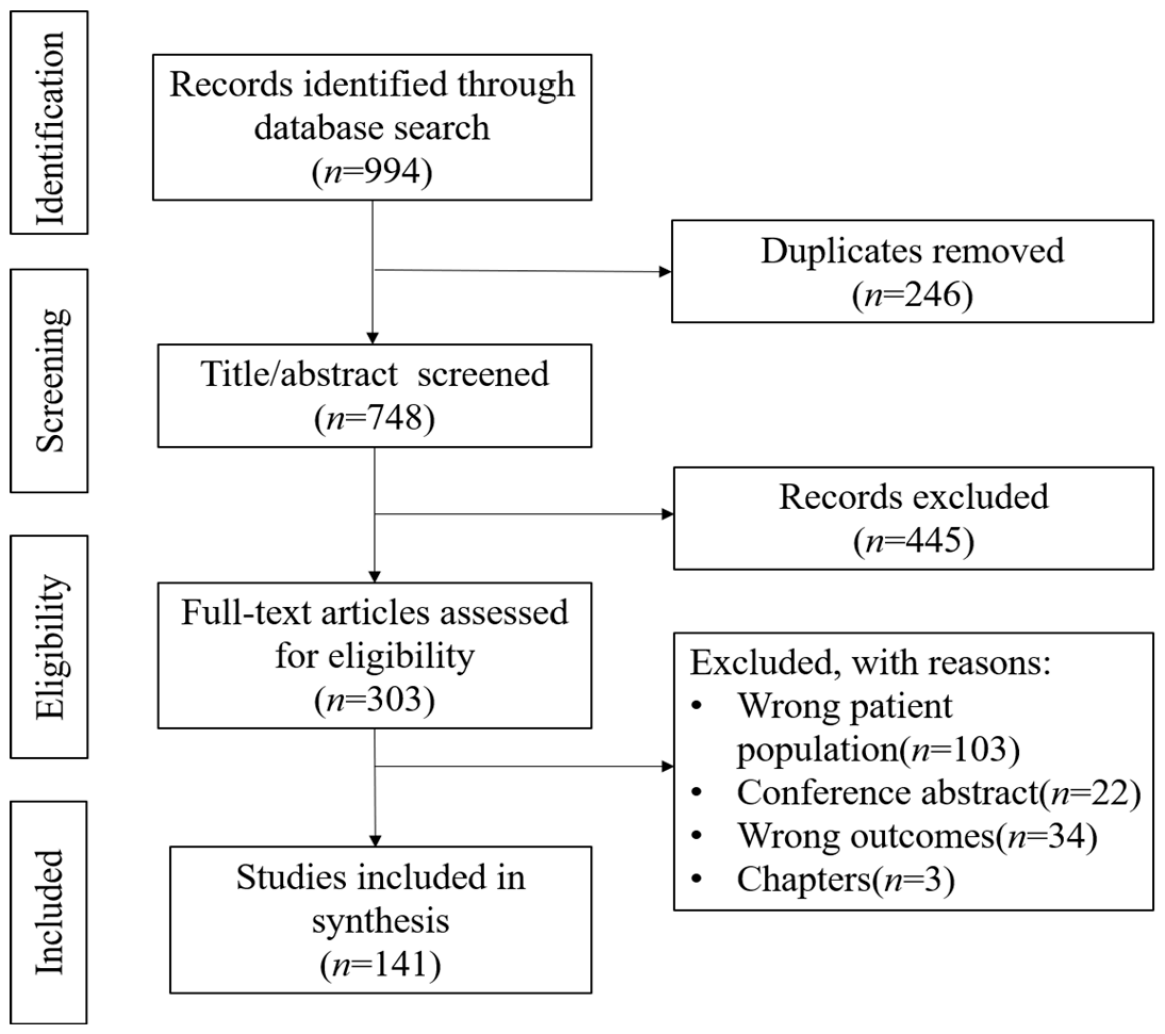
PRISMA-standardized flow diagram of literature screening.

A comprehensive literature search was performed across the PubMed, Web of Science, Scopus and EMBASE databases, with publication dates from January 2010 to June 2026. The search strategy combined medical subject headings (MeSH/Emtree) and free-text synonyms, which were divided into three core groups: (1) exosomes and extracellular vesicles; (2) long noncoding RNAs (lncRNAs); and (3) cerebrovascular diseases and stroke. The Boolean operator “OR” was applied to integrate synonymous terms within each group, while “AND” was used to intersect the three groups to capture studies covering all three themes simultaneously. Only original research articles, systematic reviews and meta-analyses were retained; conference abstracts, letters, case reports, commentaries and duplicate records were excluded. We initially screened all the retrieved records by title and abstract and then further evaluated the full-text articles of potentially eligible studies. The reference lists of the included papers and relevant reviews were manually reviewed for supplementary eligible studies. On the basis of the literature, this review systematically describes the functional mechanisms of exosomal lncRNAs in the pathological progression of cerebrovascular diseases, as well as their translational value as novel diagnostic biomarkers and therapeutic intervention targets for stroke and cerebrovascular lesions. The study-selection process is presented in Figure 1.

## 2. Exosomes

### 2.1. Biological Characteristics of Exosomes

The biological characteristics of exosomes determine their unique role in intercellular communication and disease development [9]. A deep understanding of the fundamental biological properties of exosomes is highly important for elucidating their functional mechanisms in cerebrovascular diseases. Exosomes possess three interrelated biological characteristics. First, their biogenesis process involves complex regulatory mechanisms from their formation within the intracellular system to their eventual release. Second, there is a mechanism through which exosomes are taken up by cells and affect them. Third, the way in which exosomes travel through tissues follows a patterned distribution in space and time, and this distribution is affected by both pathology and normal physiology in various tissues throughout the body.

#### 2.1.1. Biogenesis of Exosomes

**Figure 2:**
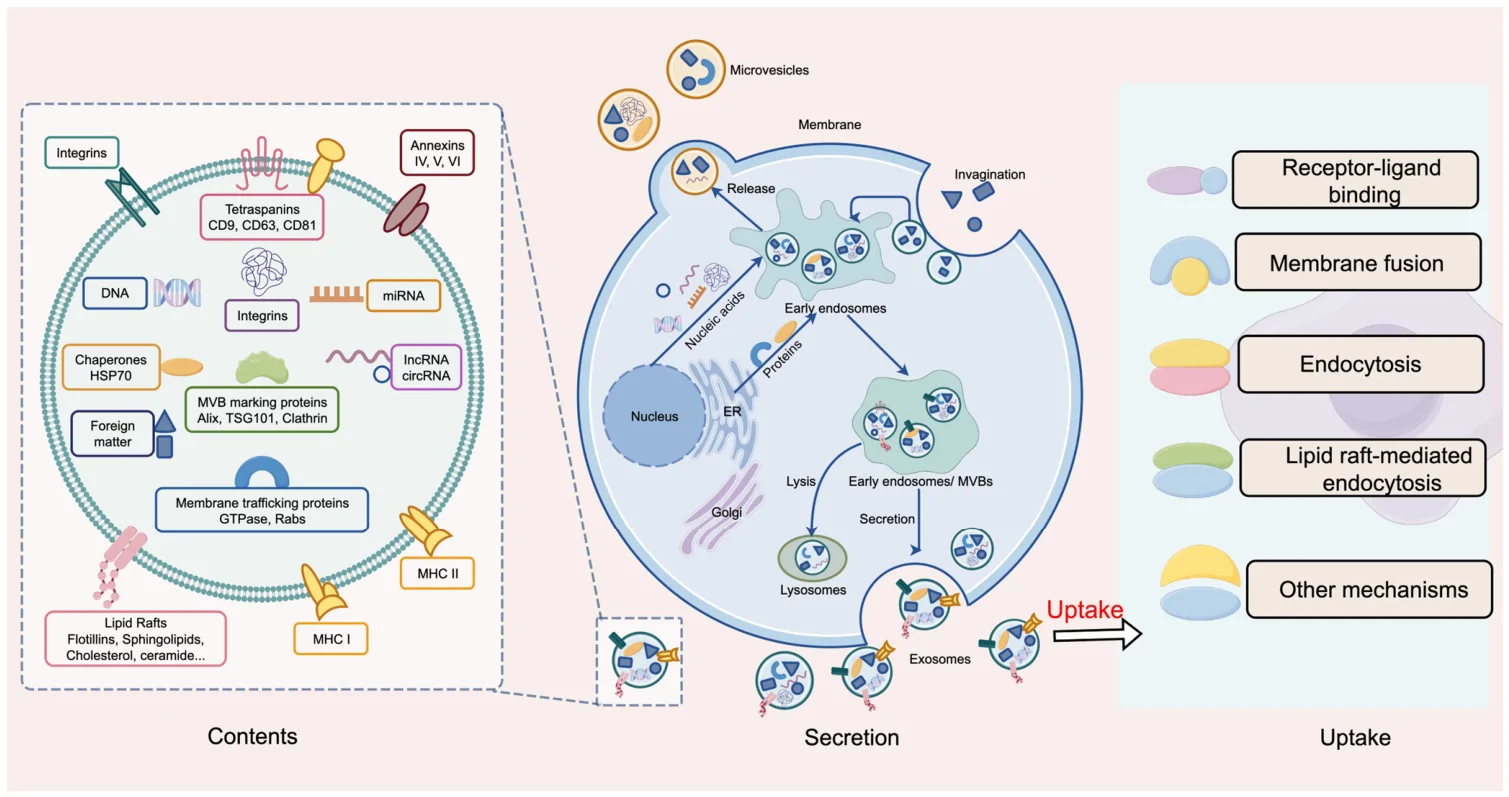
Schematic illustration of exosome biogenesis, composition, and cellular uptake mechanisms. Left panel (Contents): Exosomes contain diverse bioactive molecules, including membrane proteins (tetraspanins CD9, CD63, and CD81; integrins; major histocompatibility complex (MHC) class I and II molecules; annexins IV, V, and VI), lipid raft components (flotillins, sphingolipids, and chaperones (heat shock protein 70, HSP70)), MVB-associated proteins (Alix, TSG101, clathrin), membrane trafficking regulators (GTPases, and Rabs), and nucleic acids (DNA, miRNA, lncRNA, circRNA). Middle panel (Secretion): Exosome biogenesis begins with plasma membrane invagination to form early endosomes, which mature into multivesicular bodies (MVBs) containing intraluminal vesicles. MVBs fuse either with lysosomes for degradation or with the plasma membrane to release exosomes into the extracellular space. Right panel (Uptake): Recipient cells internalize exosomes through multiple mechanisms.

Exosomes are double-membrane vesicles with a density of 1.10–1.19 g/mL that selectively transport biomolecules such as proteins, nucleic acids, and lipids [10]. They were first identified in sheep reticulocytes by Pan et al. [11]. Later in 1983, the term “exosomes” was coined by Johnstone et al. [12]. As illustrated in the middle panel of Figure 2, exosome biogenesis begins with invagination of the plasma membrane to form early endosomes. During endosomal maturation, nucleic acids and proteins are sorted into the endosomal lumen, while the endosomal sorting complexes required for transport (ESCRT) machinery drives the inward budding of the endosomal membrane to generate intraluminal vesicles (ILVs) [13,14]. The progressive accumulation of ILVs gives rise to multivesicular bodies (MVBs) [15]. Mature MVBs then have one of two fates: they can fuse with lysosomes for degradation, or with Rab GTPase- and soluble *N*-ethylmaleimide-sensitive factor attachment protein receptor (SNARE) protein-directed transport to the plasma membrane, where they fuse and release ILVs as exosomes into the extracellular space [16].

#### 2.1.2. Mechanisms of Exosome Uptake by Cells

After secretion, exosomes are internalized by recipient cells through various mechanisms to mediate intercellular communication [17]. As shown in the right panel of Figure 2, five principal uptake routes have been characterized: (1) receptor–ligand binding: exosomal transmembrane ligands engage cognate receptors on recipient cells, activating downstream signaling pathways that modulate cellular functions; (2) membrane fusion: Exosomes directly fuse with the plasma membrane of recipient cells, releasing encapsulated bioactive molecules into the cytoplasm for rapid functional regulation; (3) endocytosis: This is the predominant uptake mechanism, including clathrin-mediated and caveolin-mediated pathways, through which exosomes are internalized into endosomal compartments for intracellular processing [18,19,20]; (4) lipid raft-mediated endocytosis: exosomal membrane components specifically bind to cholesterol- and sphingolipid-enriched lipid raft microdomains, triggering membrane invagination and vesicle internalization via a cholesterol-dependent mechanism [18]; and (5) other mechanisms, including phagocytosis and macropinocytosis, which are primarily employed by macrophages and dendritic cells for nonselective exosome engulfment and play important roles in immune regulation and clearance of circulating exosomes [21,22]. The selection among these routes depends on the exosome source, recipient cell type, surface molecular composition, and local microenvironmental conditions.

#### 2.1.3. Tissue Distribution of Exosomes

Exosomes represent an essential pathway of communication between cells; they can be found throughout the body, including in blood and cerebrospinal fluid, and they vary depending on where they are produced and what diseases they help to characterize. The quantity and molecular patterns of exosomes have been demonstrated to reflect the state of cerebrovascular disease. Heterogeneous central nervous system (CNS)-cell-derived interleukin-6 (IL-6) in cerebrospinal fluid-derived small extracellular vesicles (sEVs) is significantly correlated with clinical severity in aneurysmal subarachnoid hemorrhage (aSAH) and is considered a potential biomarker for evaluating aSAH evolution [23]. In subacute stroke patients, changes in the number of platelet-derived exosomes (PVs) and neuron-derived exosomes (NVs) in peripheral blood are closely related to the degree of functional recovery six months later, where lower levels of PV and NV indicate poorer long-term neurological recovery [24]. Importantly, brain-derived exosomes traverse the blood–brain barrier to enter the bloodstream through specific mechanisms. The cerebrospinal fluid exosomes of Alzheimer’s disease patients contain abnormal levels of marker proteins such as Aβ42, Aβ42/40, Tau, and P-T181-Tau, and these changes can be simultaneously observed in peripheral blood, providing new insights for the early diagnosis of neurodegenerative diseases [25].

The in vivo distribution of exosomes is precisely regulated by multiple factors. First, variations in the surface proteins and lipid composition result in tissue targeting. OM-MSC-derived A2M-AS1 ameliorates oxidative stress via regulation by IGF2BP1-targeted TP53INP1, inducing mitophagy [26]. Second, local microenvironmental conditions also strongly influence the distribution of exosomes. In cerebrovascular disease, hypoxia is one type of microenvironmental change and can induce changes in the expression of many lncRNAs in exosomes. Hypoxia increases the expression of the lncRNA MEG3, which is involved in neuronal cell death and the inhibition of angiogenesis [27]. The expression of lncRNA H19 is also increased under hypoxic conditions, and it can inhibit the process of autophagy through the DUSP5–ERK1/2 axis, aggravating neural damage [28]. Moreover, the expression of the lncRNA Tug1 is increased under inflammatory microenvironmental conditions, and Tug1 can further regulate microglial pyroptosis by inhibiting PINK1/Parkin-mediated mitophagy [29]. The lncRNA Maclpil, which is preferentially expressed in macrophages, participates in inflammatory regulation; siRNA-mediated knockdown of Maclpil reduces ischemic injury and improves neurological function [30]. Blood–brain barrier integrity is also an important factor influencing the distribution of exosomal lncRNAs. lncRNA-p21 is downregulated in brain microvascular endothelial cells, but lncRNA-p21 overexpression can prevent autophagy-dependent degradation of occludin through the PI3K/AKT/mTOR signaling pathway, thus preserving the structure and functional stability of the blood–brain barrier. In contrast, the expression of the lncRNA FAL1 is downregulated under hypoxia–reoxygenation conditions, and overexpression of the lncRNA FAL1 can protect brain microvascular endothelial cells from injury by regulating PAK1 [31].

### 2.2. Functions of Exosomes

#### 2.2.1. Intercellular Communication

Exosomes act as bioactive components for transmitting signaling molecules (such as proteins, lipids, and nucleic acids derived from donor cells) as part of cellular communication. When dendritic cell-derived exosomes transport MHC–antigen complexes, for example, T cells are formed and an adaptive immune response develops [32]. Other areas where exosomes are implicated as agents of intercellular signal communication include the nervous system. Studies on microglia-derived exosomes have provided evidence of how microglia-derived exosomes transport specific forms of proinflammatory cytokines, as well as α-synuclein (α-syn) oligomers, in models of Parkinson’s disease (PD), indirectly stimulating the expression of the E3 ubiquitin ligase Peli1 in astrocytes. Peli1 expressed by these astrocytes further directs those astrocytes toward neurotoxic A1 phenotypes, thus creating adipogenic signaling loops that further injure to dopaminergic neurons [33]. Accumulating evidence has demonstrated that exosomes derived from proinflammatory microglia potently activate the NLRP3 inflammasome, thereby upregulating the expression of ASC, pro-caspase-1, and caspase-1, which ultimately aggravates α-synuclein-induced neuronal damage [34].

**Figure 3:**
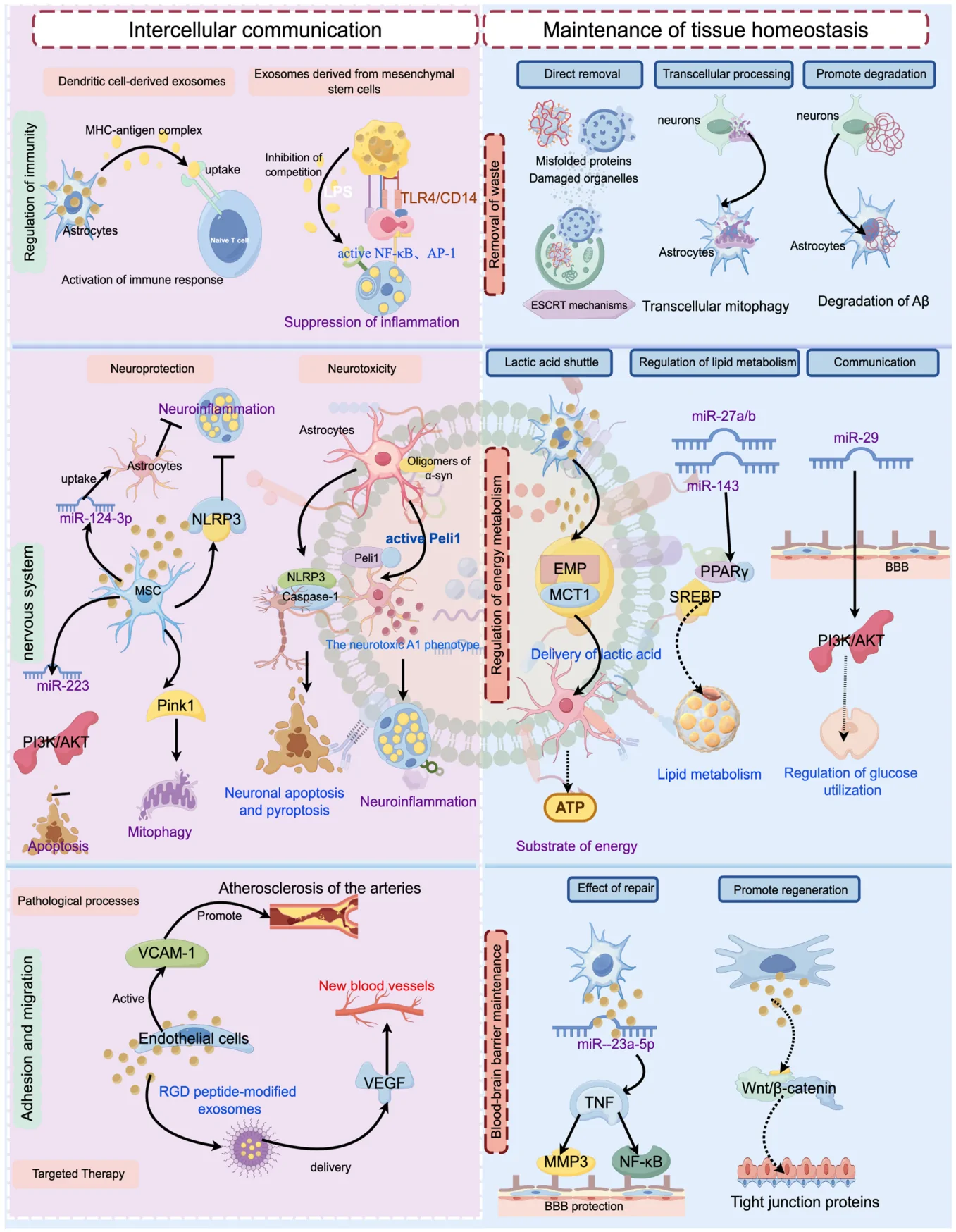
Multifaceted physiological functions of exosomes in the central nervous system and vasculature. Exosomes mediate intercellular communication and maintain tissue homeostasis through the regulation of immunity, nervous system function, cell adhesion and migration, waste removal, energy metabolism, and blood–brain barrier integrity. MSC, mesenchymal stem cell; LPS, lipopolysaccharide; TLR4, Toll-like receptor 4; NF-κB, nuclear factor kappa-B; AP-1, activator protein-1; NLRP3, NOD-like receptor protein 3; Pink1, PTEN-induced kinase 1; PI3K, phosphoinositide 3-kinase; Akt, protein kinase B; VCAM-1, vascular cell adhesion molecule-1; VEGF, vascular endothelial growth factor; RGD, arginine–glycine–aspartate; ESCRT, endosomal sorting complex required for transport; Aβ, amyloid-beta; EMP, Embden–Meyerhof pathway; MCT1, monocarboxylate transporter 1; ATP, adenosine triphosphate; PPARγ, peroxisome proliferator-activated receptor gamma; SREBP, sterol regulatory element-binding protein; BBB, blood–brain barrier; TNF, tumor necrosis factor; MMP3, matrix metalloproteinase 3; α-syn, alpha-synuclein; Solid-line arrows: direct effects; dashed-line arrows: indirect effects.

The exosomes from mesenchymal stem cells (MSC-Exos) have been shown to exert neuroprotective qualities in exosome-derived research. MSC-Exos inhibit the binding of lipopolysaccharide (LPS) to Toll-like receptor 4 (TLR4) via the TLR4/CD14 complex, downregulate IκBα and AP-1 transcription, reduce the release of proinflammatory factors, and alleviate neuroinflammation [35]. The delivery of miR-223 by MSC-Exos activates the PI3K/Akt signaling pathway to reduce hypoxia-induced neuronal apoptosis, and improve neuroinflammation in cerebral ischemia–reperfusion injury [36]. MSC-Exos are involved in regulating mitophagy. Astrocyte-derived exosomes activate Pink1-mediated mitophagy, which clears damaged mitochondria, inhibits NLRP3 inflammasome activation, reduces the release of IL-1β and ASC, suppresses oxidative stress injury, and improves Parkinson’s disease outcomes [37]. Neuron-derived exosomes containing miR-124-3p can be internalized by astrocytes, inhibiting the activation of M1 microglia and A1 astrocytes, thereby promoting functional behavior recovery and protecting against spinal cord injury [38]. (A schematic diagram of exosome-mediated intercellular communication is shown on the left side of Figure 3.)

Another major role of exosomes in intercellular communication is cell adhesion and migration behavior. Exosomes derived from endothelial cells (EDEs) found in plasma of atherogenic individuals express elevated levels of VCAM-1, which promotes monocyte adhesion to the vascular endothelium, leading to lipid deposition and plaque formation [39]. In addition, engineered exosomes can be used for specific targeting of cells. The insertion of arginine–glycine–aspartate (RGD) peptides into extracellular vesicles (EVs) allowed them to specifically recognize and fuse with integrin αVβ3, which was overexpressed in the ischemic area, increasing their enrichment efficiency at diseased vascular sites. These modified exosomes not only increase the target recognition of vascular endothelial cells but also release vascular endothelial growth factor (VEGF) to promote angiogenesis and neural function recovery [40]. (A schematic diagram of exosome-mediated intercellular communication is shown in the lower-left section of Figure 3.)

#### 2.2.2. Maintaining Tissue Homeostasis

Tissue homeostasis is the basis of internal environmental stability. An imbalance in tissue homeostasis contributes to the occurrence and development of many diseases. As important mediators of the intercellular communication process, extracellular vesicles play important roles in tissue homeostasis, especially in neurovascular unit function, mainly through two pathways: metabolic waste clearance and energy metabolism regulation.

Exosome-mediated transcellular waste clearance helps maintain intracellular homeostasis. Damaged or misfolded proteins and organelles occur naturally in cellular metabolism, but may cause cellular dysfunction or death if they are not removed. Exosomes participate in this process through multiple pathways. Exosomes are involved in the direct clearance pathway in which the ESCRT complex recognizes and sorts misfolded proteins into intraluminal vesicles during the formation of multivesicular bodies that are released extracellularly as exosomes during exocytosis [41]. Neuron-derived exosomes contain amyloid-beta (Aβ) oligomers and phosphorylated tau proteins and can reduce the load of intracellularly toxic proteins. However, this mechanism has a dual nature: if they are not eliminated quickly, exosomes can release these proteins, allowing them to spread into neighboring cells [42]. Exosomes also contribute to the phagocytic function of target cells through signaling. Monocytes and endothelial cells can be activated through TLR pathways by exosomes, resulting in a cascade of inflammatory response mediators [43,44]. Exosomes produced from neurons in an Alzheimer’s disease model produce an altered form of Aβ that is less toxic and more efficiently taken up by microglia and then degraded by lysosomal processes [45]. Additionally, the complexity of the exosome-mediated system for transcellular waste elimination stems from the fact that the process also involves the uptake of damaged organelles by other cell types. Damaged mitochondria can be incorporated into exosomes via “mitochondria-derived vesicles” and taken up and degraded by nearby astrocytes, providing neurons with a unique organelle quality control system [46]. (See the schematic diagram of exosome-mediated homeostasis maintenance in the upper-right part of Figure 3.)

During energy metabolism regulation, exosomes can be called “metabolic messengers” as they coordinate the energy supply and demand balance between all cell types that compose the neurovascular unit. The human brain accounts for only 2% of the total body weight but a full 20% of the energy usage by the entire body. Thus, intercellular metabolic cooperation becomes extremely important. In the regulation of glucose metabolism, exosomes derived from astrocytes are rich in monocarboxylate transporters as well as key glycolytic enzymes such as lactate dehydrogenase and hexokinase 2. Under conditions of energy stress, exosomes are critical enhancers of the “astrocyte–neuron–lactate shuttle” [47,48]. Additionally, exosomes secreted by pancreatic β-cells contain miRNAs belonging to the miR-29 family, which cross the blood–brain barrier to regulate insulin/PI3K/Akt signaling pathways and thus glucose uptake and usage by the brain. This mechanism is particularly important for cognitive impairment in people with diabetes [49]. Exosome-mediated intercellular communication also plays an important role in regulating lipid metabolism. Under oxidative stress, exosomes released by visceral adipocytes carry regulatory molecules such as miR-27a/b and miR-143, which promote a shift in the lipid metabolism pattern of recipient cells from synthesis to catabolism by downregulating PPARγ and SREBP1/2 expression, thereby increasing fatty acid β-oxidation to address energy crises [50]. In addition, reverse cholesterol transport involving exosomes is crucial for maintaining cholesterol homeostasis in the brain and the integrity of myelin. Interestingly, exosomal glutaminase can also regulate the activity of the glutamine–glutamate cycle within astrocytes, hence indirectly affecting the production of neurotransmitters and the excitability of neurons [51]. (See the schematic diagram of exosome-mediated homeostasis maintenance in the middle-right part of Figure 3.)

In the pathological process of cerebrovascular diseases, the homeostatic regulatory function of exosomes is complex and bidirectional. Exosomes secreted from the brain endothelial cells during the acute phase of ischemia-induced stroke can induce microglial reprogramming to the proinflammatory M1 phenotype (which promotes inflammatory activity), and thus cause further damage to the nervous system and vasculature [52]. However, Rab27a-dependent exosomes exert neuroprotective effects by activating the antioxidant defense system, and reducing oxidative stress injury and apoptosis in endothelial cells [53]. The dual role of exosomes depends upon both the condition of the donor (i.e., the brain endothelial cells) and the microenvironment in which the exosomes are located. When the integrity of the blood–brain barrier is impaired, exosomes have great potential for repair. Extracellular vesicles (EVs) from M2 microglia reduce postischemic blood–brain barrier disruption through the delivery of miR-23a-5p, a mechanism associated with the targeting of TNF by miR-23a-5p and the regulation of the expression of MMP3 and NFκB p65 [54]. Exosomes produced from neural stem cells (EVs-WK) induce the proliferation and angiogenesis of endothelial cells through the activation of the Wnt/β-catenin pathway and increase the number of tight junctions formed between endothelial cells, thereby increasing perfusion in the brain and maintaining the structural integrity of the blood–brain barrier [55]. (See the schematic diagram of exosome-mediated homeostasis maintenance in the lower-right section of Figure 3.)

### 2.3. Isolation and Extraction of Exosomes

Exosomes are nanosized extracellular vesicles that are ubiquitous in biological fluids. Their tiny size and low density make them vulnerable to contamination by endogenous proteins, lipids and nucleic acids, severely compromising experimental reproducibility. In accordance with the ISEV’s cumulative MISEV guidelines spanning 2013–2023, uniform isolation procedures and cross-verification are essential for credible exosome research, as no one purification method can fit all experimental scenarios [56]. Three mainstream techniques with distinct advantages and disadvantages are widely adopted. Ultracentrifugation, the classic gold-standard method, purifies exosomes on the basis of density and differences in the sedimentation coefficient. Stepwise centrifugation removes debris and large vesicles, yielding reagent-free, bioactive exosomes with broad sample compatibility, despite low recovery and time-consuming operation [57]. PEG-based polymer precipitation enables fast, low-cost and high-throughput extraction without sophisticated instruments, and is suitable for large-scale preliminary screening, yet its low purity and residual polymer interference limit functional assays [58]. Immunoaffinity capture specifically enriches exosomes via CD9/CD63/CD81 antibody-modified beads for ultrahigh purity [59]. Size-exclusion chromatography (SEC) separates exosomes from soluble proteins on the basis of molecular size under gravity flow, preserving vesicle structural integrity and biological activity, although it dilutes samples and cannot distinguish exosomes from similarly sized microvesicles. Emerging microfluidic platforms exploit laminar flow and microscale forces to achieve rapid, high-purity exosome isolation with minimal sample consumption, yet their limited throughput and lack of standardized scalability currently restrict large-scale clinical application. In accordance with the MISEV2024 recommendations, orthogonal validation combining multiple isolation methods is strongly encouraged to eliminate technical bias and improve the credibility of results.

## 3. Overview of LncRNA

Long noncoding RNAs were first defined as transcripts of longer than 200 nucleotides with little or no protein-coding ability. The Human Genome Project and the subsequent ENCODE project revealed that more than 90% of the human genome is transcribed, but only 1–2% corresponds to protein-coding genes [60]. Among the vast array of noncoding transcripts, lncRNAs have attracted attention because of their abundance and the difficulty of defining their functionalities. Substantial evidence suggests that lncRNA expression is highly spatiotemporally specific and plays critical roles in life activities such as development, differentiation and disease [61]. Although the primary sequence of lncRNAs is less conserved than that of mRNAs, the secondary and tertiary conserved structures and their specific interactions with DNA/RNA/protein dictate their bioprocesses, which differ from those of mRNAs [62]. This discovery expands the central dogma of molecular biology and provides a deep insight into the underlying principles of the functional complexity of gene expression regulatory networks.

The functional mechanisms of lncRNAs are diverse and precise. In terms of their molecular mode of action, lncRNAs can function as molecular signals, molecular decoys, molecular guides, or molecular scaffolds to regulate gene expression at various levels, such as the epigenetic, transcriptional, and posttranscriptional levels [63]. These key mechanisms provide a molecular basis for the involvement of lncRNAs in other diseases, such as cerebrovascular diseases. In terms of epigenetic regulation, lncRNAs perform accurate chromatin modification. Xist (X-inactive specific transcript) was reported to inactivate the entire X chromosome by coating the X chromosome and recruiting chromatin-modifying complexes, including histone methyltransferases and DNA methyltransferases [64]. Similarly, lncRNAs such as H19 and Kcnq1ot1 silence the specific regions caused by binding to Polycomb Repressive Complex 2 (PRC2), which in turn catalytically trimethylates lysine 27 of histone H3 (H3K27me3) [65]. This molecular scaffold function endows lncRNAs with the ability to deliver chromatin-modifying complexes to specific gene loci, and plays a key role in cell differentiation and fate decisions.

Transcriptional regulation is another important functional domain of lncRNAs. LncRNAs that are located near a promoter can influence the transcription of that promoter region (cis action) or act through their interaction with other promoters (trans action) depending on whether they impact the binding of transcription factors to DNA and/or change the local chromatin arrangement [66]. Moreover, some lncRNAs act by sequestering transcription factors, preventing them from binding to target DNA sequences. In the DNA damage response, the lncRNA PANDA maintains cell survival by binding to the nuclear transcription factor NF-YA and inhibiting the activation of proapoptotic genes [67].

Transcriptional regulation is another function of lncRNAs. In the cytoplasm, lncRNAs regulate the fate of mRNAs through various mechanisms. With respect to competitively expressed RNAs (ceRNAs), lncRNAs can act as molecular sponges that absorb individual miRNAs, alleviating their inhibitory effects on target mRNAs and leading to the formation of complex ceRNA regulatory networks [68]. In addition to playing a role in epigenetic regulation, some types of HOTAIR act as ceRNAs in the cytoplasm to regulate the expression of genes involved in cell growth and migration. In addition, lncRNAs can regulate mRNA stability by forming RNA–RNA duplexes or regulate translation efficiency by binding to RNA-binding proteins. The roles of lncRNAs extend to protein regulation and cellular structural organization. MALAT1 precisely regulates the alternative splicing of precursor mRNAs by interacting with serine/arginine-rich splicing factors [69]. At the subcellular level, NEAT1, which is essential for paraspeckle formation, is essential for the cellular stress response through the sequestration of specific transcripts and proteins.

## 4. Excellent Clinical Application Potential of Exosomal lncRNAs

Exosomes contain lncRNAs as part of providing natural intercellular communication and have shown promise in both the diagnosis and treatment of cerebrovascular diseases. The lipid bilayer component of exosomes forms a protective barrier around the lncRNA components within, allowing greater protection against RNases in the circulation and thus increasing stability within all body fluids, including blood and cerebrospinal fluid (CSF). Furthermore, the level of stability of lncRNAs in exosomes is much greater than that of freely circulating lncRNAs [70]. Although lncRNA expression levels are low in certain cells, they are highly enriched in the exosomes secreted by these cells. This selective packaging mechanism is closely related to disease progression [71]. Exosomes can be effectively isolated and purified using a variety of well-established methods including but not limited to differential ultracentrifugation, polymer precipitation, and immunoaffinity capture [71,72].

Exosomal lncRNAs from different cellular sources play important regulatory roles in cerebrovascular diseases (see Table 1). Exosomal lncRNAs derived from mesenchymal stem cells (MSCs) exhibit significant neuroprotective effects. Among them, lncKLF3-AS1 functions through multiple molecular mechanisms: In cerebral ischemia–reperfusion injury, MSC-derived exosomes carrying lncKLF3-AS1 inhibit neuronal apoptosis by downregulating the YY1/MSI1 axis and regulating the Sphk1/NF-κB pathway [73]. Moreover, this lncRNA reduces cerebral infarct volume and improves neurological function through the miR-206/USP22 axis [74]. Additionally, exosomal lncZFAS1 secreted by bone marrow MSCs effectively alleviates MCAO-induced oxidative stress, cerebral infarction, and inflammation through binding to miR-15a-5p [75]. Exosomal lncOIP5-AS1 from M2 microglia reduces neuronal pyroptosis and improves cerebral ischemia–reperfusion injury by inhibiting TXNIP protein expression [76].

Exosomal lncRNAs have great potential for clinical translation because they may be easily acquired through non or minimally invasive procedures such as liquid biopsy. As a result, exosomal lncRNAs can be used for large-scale screening and dynamic monitoring of patients [77]. Another key feature of exosomes is their ability to cross the blood–brain barrier, which makes them an excellent vehicle for delivering targeted therapies to patients with central nervous system disorders. By using one of several different methods (e.g., drug pretreatment of donor cells, ultrasound to increase membrane permeability, or lentiviral-mediated modification of exosomal genes), it is possible to significantly enhance the effects of exosome-based therapies [78]. Buyang Huanwu decoction can regulate the exosomal MALAT1/YAP1/HIF-1α axis via the endocytosis-related protein caveolin-1 (Cav1), promoting angiogenesis after cerebral ischemia [79]; macrophage migration inhibitory factor-pretreated MSC-derived exosomal lncNEAT1 regulates oxidative stress and inhibits H_2_O_2_-induced cell apoptosis through the miR-142-3p/FOXO1 axis [80]. These preclinical findings suggest potential diagnostic and therapeutic applications that require further clinical validation before translation into routine practice.

**Table 1:** Therapeutic effects of exosomal lncRNAs from different cell sources in cerebrovascular diseases.

Source	LncRNA	Expression	Target/Mechanism	Experimental Models	Functional Impact	Ref
MSC	lncKLF3-AS1	Downregulated	YY1/MSI1/Sphk1/NF-κB	OGD/R model of MSCs and MCAO/R model in mice	Inhibits neuronal apoptosis and ameliorates cerebral ischemia–reperfusion injury	[73]
BMSC	lncKLF3-AS1	Downregulated	miR-206/USP22	BV2 microglia and MCAO/R model in mice	Reduces cerebral infarct volume and improves neurological function in MCAO mice	[74]
BMSC	lncZFAS1	Downregulated	Sponge miR-15a-5p	Exosomes derived from BMSCs intervene BV2 microglia and MCAO/R model in mice	Reduces MCAO-induced oxidative stress and inflammation	[75]
M2 Microglia	lncOIP5-AS1	Downregulated	ITCH-induces TXNIP degradation	Exosomes secreted by M2 macrophages were applied to the mouse MCAO/R model	Inhibits neuronal pyroptosis and attenuates CIRI	[76]
Buyang Huanwu-treated exosomes	lncMALAT1	Downregulated	Cav1/MALAT1/YAP1/HIF-1α pathway	MCAO/R model in Cav1-knockout mice	Promotes angiogenesis in MCAO mice	[79]
IA-PLAS	lncATP1A1-AS1	Upregulated	VSMC phenotypic switching	Exosomal lncRNAs isolated from patients with intracranial aneurysms were investigated in vascular smooth muscle cells	Induces VSMC phenotype switching and apoptosis; Upregulates MMP-9	[81]
ox-LDL- HUVEC	lncCLDN10-AS1	Upregulated	miR-186/YY1 axis	Human umbilical vein endothelial cells (HUVECs) treated with oxidized low-density lipoprotein (ox-LDL)	Aggravates oxidative stress injury in vascular endothelial cells	[82]
LAA-PLAS	lnc002015	Upregulated	Sponges miR-342	Exosomes derived from patients	Modulates atherosclerosis-related inflammation	[83]
IS-PLAS	lncH19	Upregulated	let-7a/IGF1R axis	Serum exosomes from patients with ischemic stroke and mouse MCAO/R model	Downregulates IGF-1 and attenuates neuroprotective effects	[84]
SAH-PLAS	lncTM7SF3-AU1	Upregulated	miR-702-3p/SARM1 pathway	Exosomes derived from SAH models; knockout of TM7SF3-AU1 alleviates brain injury	Alleviates white matter damage and neurological deficits	[85]
THP-1	lncGAS5	Upregulated	Regulates apoptosis-related proteins	Atherosclerosis-derived sources and mechanistic investigation	Promotes endothelial apoptosis	[86]
OM-MSC	lncA2M-AS1	Upregulated	IGF2BP1/TP53INP1	MPP^+^-stimulated HT22 cells and MPTP-induced PD model in C57BL/6 mice	Attenuates oxidative stress through IGF2BP1/TP53INP1-dependent mitophagy	[26]
MSC	LOC100129516	Upregulated	PPARγ/LXRα/ABCA1 pathway	ApoE-knockout mice with atherosclerosis	Promotes cholesterol efflux and alleviates atherosclerosis	[87]
macrophage	lncPVT1	Upregulated	Sponges miR-186-5p to regulate HMGB1	AAA model constructed with Ang-II-treated VSMCs	Promotes inflammation and pyroptosis; accelerates abdominal aortic aneurysm progression	[88]

Abbreviations: MSC, mesenchymal stem cell; BMSC, bone marrow mesenchymal stem cell; MCAO, middle cerebral artery occlusion; CIRI, cerebral ischemia–reperfusion injury; IA, intracranial aneurysm; VSMC, vascular smooth muscle cell; ox-LDL, oxidized low-density lipoprotein; HUVEC, human umbilical vein endothelial cell; LAA, large artery atherosclerosis; IS, ischemic stroke; SAH, subarachnoid hemorrhage. IA-PLAS, plasma from intracranial aneurysm patients; LAA-PLAS, plasma from large artery atherosclerosis patients; IS-PLAS, plasma from ischemic stroke patients; SAH-PLAS, plasma from subarachnoid hemorrhage patients; THP-1, human monocytic leukemia cell line; OM-MSC, olfactory mucosa mesenchymal stem cells ITCH, itchy E3 ubiquitin protein ligase; TXNIP, thioredoxin-interacting protein; HMGB1, high-mobility group box 1; YY1, Yin Yang 1; MSI1, Musashi-1; USP22, ubiquitin-specific protease 22; Sphk1, sphingosine kinase 1; Cav1, caveolin-1; HIF-1α, hypoxia-inducible factor 1-alpha; YAP1, Yes-associated protein 1. AAA, abdominal aortic aneurysm.

## 5. Correlation Between Exosomal lncRNAs and Cerebrovascular Diseases

### 5.1. Exosomal lncRNAs and Oxidative Stress

As a major pathological feature of cerebrovascular diseases, oxidative stress mainly originates from the imbalance between the production of reactive oxygen species (ROS) and reactive nitrogen species (RNS) and the body’s ability to scavenge them, which results in an imbalance in redox homeostasis [89]. In cerebrovascular diseases, mitochondrial dysfunction results in the excessive production of free radicals such as superoxide anion (O_2_^−^) and hydrogen peroxide (H_2_O_2_) and decreases the activity of antioxidant enzymes such as superoxide dismutase (SOD) and glutathione peroxidase (GPx), which exacerbates the accumulation of ROS [8]. Abnormal iron metabolism leads to the production of hydroxyl radicals (·OH) by the Fenton reaction, which can induce ferroptosis in neurons [90]. The NF-κB pathway can be activated by ROS via proinflammatory cytokines such as IL-1β and TNF-α to initiate an “oxidant–inflammatory” cascade. The combination of these pathways leads to disruption of the blood–brain barrier and its associated endothelial cell dysfunction; therefore, ultimately increased neuronal apoptosis may occur, resulting in greater damage to the brain.

Exosomal lncRNAs can regulate the effects of oxidative stress on target tissues through their selective packaging and distribution to specific target cells. BMSC-derived exosomal lncRNA ZFAS1 upregulates SOD expression by sponging miR-15a-5p, significantly reducing MDA levels and oxidative stress damage in MCAO model mice [75]. Exosomal lncKLF3-AS1 activates the Sphk1 signaling through the YY1/MSI1 axis, inhibiting NF-κB-mediated ROS production and alleviating OGD/R-induced neuronal damage [73]. Other exosomal lncRNAs, such as A2M-AS1, promote mitophagy, clearing damaged mitochondria and reducing ROS production via the IGF2BP1/TP53INP1 signaling axis [26]. Exosomal lncRNAs can also indirectly regulate oxidative stress by inducing metabolic reprogramming in target tissues. VIM-AS1 activates the PPAR-γ pathway to promote glycolysis, thereby increasing ATP synthesis for cell repair and decreasing oxidative phosphorylation-related ROS production [91]. Multiple exosomal lncRNAs relieve cerebrovascular neuronal damage by regulating oxidative stress, supporting mechanistic research and targeted treatment. However, most studies rely on cell and animal models and lack clinical evidence; further research is needed to determine their dosage effects, long-term mechanisms and clinical safety.

### 5.2. Exosomal lncRNAs and Apoptosis

Apoptosis, a key pathological process in cerebrovascular diseases, is essentially a programmed cell death process that is strictly regulated by multiple genes. Its underlying mechanisms occur primarily through three classical pathways [92]: the mitochondrial pathway (intrinsic pathway), whose effect is primarily through enhancing mitochondrial outer membrane permeability (MOMP) to facilitate the release of cytochrome C (Cyt c) into the cytoplasm, which subsequently binds to Apaf-1 to template the apoptosome and activate the Caspase-9 cascade; the death receptor pathway (extrinsic pathway), in which death receptors (e.g., Fas and TNF-R1) are programmed to bind to their ligands and recruit FADD and Caspase-8 to form the death-inducing signaling complex (DISC); and the endoplasmic reticulum stress pathway, which facilitates the activation of CHOP and JNK signaling via the unfolded protein response (UPR) to accelerate apoptosis. These processes of apoptosis are accompanied by the production of numerous inflammasomes and the activation of proinflammatory factors, such as IL-1β and IL-18, which can lead to neuronal damage, disruption of the blood–brain barrier, and endothelial dysfunction, further aggravating damage to brain tissues.

Exosomal lncRNAs play important regulatory roles in the development of cerebrovascular diseases by specifically altering apoptosis-related pathways. BMSC-derived KLF3-AS1 activates the Sphk1/NF-κB pathway via the YY1/Musashi-1 axis, thereby reducing the levels of proinflammatory cytokines (IL-6 and TNF-α) in a mouse model of MCAO-induced neuronal injury [73]. KLF3-AS1 also upregulates USP22 expression by sponging miR-206, thereby inhibiting Sirt1 ubiquitination and degradation, prolonging its half-life, and exerting antiapoptotic effects [74]. In contrast, certain exosomal lncRNAs promote apoptosis: the expression of the lncRNA ATP1A1-AS1 is significantly upregulated in the plasma exosomes of patients with intracranial aneurysms, and its overexpression induces vascular smooth muscle cell phenotype switching, promotes apoptosis, and upregulates the expression of matrix metalloproteinase-9 (MMP-9) [81]; CLDN10-AS1, in ox-LDL-induced vascular endothelial injury, upregulates YY1 expression by sponging miR-186, thereby exacerbating apoptosis [82]. Exosomal lncRNAs regulate cerebrovascular disease bidirectionally by modulating apoptosis pathways, offering novel targets to block neuronal apoptosis. However, most related studies are limited to cell and animal models. The dual regulatory thresholds of various lncRNAs are unclear, restricting clinical targeted therapy.

### 5.3. Exosomal lncRNAs and the Inflammatory Response

The inflammatory response, as a core component of the pathophysiological process of cerebrovascular diseases, occurs throughout the entire course of neural injury, blood–brain barrier disruption, and tissue repair and remodeling [7]. During acute ischemic stroke, the innate immune response is induced by hypoxia and ischemia, resulting in the transdifferentiation of microglia from a resting phenotype to an activated phenotype. Moreover, neutrophils and monocytes/macrophages in the periphery are attracted to and infiltrate the brain parenchyma through the damaged blood–brain barrier, where they secrete proinflammatory polypeptides such as IL-1β, IL-6, and TNF-α, which aggravate neuronal death. In cerebral hemorrhage, toxic substances released from the hematoma, such as hemoglobin and iron free ions, can activate TLR4 to induce ferroptosis and activate the secondary inflammatory cascade. This regulatory process involves many signaling pathways; however, the NF-κB signaling pathway is important for the regulation of inflammation, and when activated, it affects the expression of inflammation-related genes at the transcriptional level. The MAPK/PI3K/AKT pathway is the key hub of inflammatory signal transduction and can regulate the expression of inflammatory mediators such as COX-2 and iNOS, and stimulate the upregulation of the expression of the microglial markers CD68 and Iba-1 [93]. The regulation of macrophage polarization, and therefore, the inflammatory environment, can also involve the JAK2/STAT3 signaling pathway. Additionally, the inflammatory response is maintained by an increase in activity associated with the mTOR pathway, and inhibition of mTOR has been shown to decrease neuroinflammation and hypersensitivity to pain.

Exosomal lncRNAs, key mediators of intercellular communication, bidirectionally control cerebrovascular diseases through highly specific control of inflammatory signaling networks. Exosomal lncRNA functions vary according to cellular source, microenvironmental conditions, and disease stage. With respect to anti-inflammatory regulation, ZFAS1 from BMSCs significantly suppresses IL-1β, IL-6, and TNF-α expression by competitively adsorbing miR-15a-5p while upregulating SOD activity and downregulating MDA, significantly alleviating neuroinflammation and oxidative stress during the course of ischemia–reperfusion injury [75]. KLF3-AS1 promotes Sphk1 expression via the YY1-Musashi-1 axis, thereby inhibiting NF-κB pathway activation and the inflammatory response in OGD/R-induced BV-2 microglia [73]. TGF-β1-preconditioned hUCMSC-derived UCA1 suppresses microglial overactivation and inflammatory mediator secretion by sponging miR-96-5p to upregulate FOXO3a, thereby alleviating inflammatory neuropathic pain [94]. However, some exosomal lncRNAs are proinflammatory. The expression of the lncRNA COX2 is positively correlated with that of NF-κB, and the upregulation of COX2 expression reverses the anti-inflammatory effects of drugs [95]. In atherosclerotic stroke, plasma exosomal lncRNAs (lnc_002015, and lnc_001350) regulate cell adhesion molecule expression, affecting atherosclerotic plaque stability and inflammatory progression. These lncRNAs have high diagnostic value (AUC > 0.95) and may serve as disease biomarkers [83]. Collectively, exosomal lncRNAs bidirectionally regulate the neuroinflammatory response in cerebrovascular diseases through distinct anti- and proinflammatory signaling mechanisms, offering candidate biomarkers and therapeutic targets for neuroinflammation-related injury. However, most mechanistic evidence remains restricted to cell and animal models, the thresholds governing the anti- versus proinflammatory switch of individual lncRNAs are incompletely defined, and the diagnostic value of candidate biomarkers awaits validation in larger, multicenter clinical cohorts.

## 6. Exosomal lncRNAs Participate in the Pathological Process of Cerebrovascular Diseases

### 6.1. Exosomal lncRNAs and Acute Cerebrovascular Disease

Acute cerebrovascular disease refers to various diseases in which the blood vessels that supply blood to the brain become abruptly occluded or rupture, preventing blood from reaching areas of the brain normally supplied by that blocked/ruptured vessel, resulting in immediate neurological dysfunction in those affected parts of the brain. Acute ischemic stroke (approximately 60%) is the most frequent type, while intracerebral hemorrhage, subarachnoid hemorrhage and other types of hemorrhagic strokes make up the majority of the other types. A comprehensive understanding of exosomal lncRNAs may clarify the pathophysiology of acute cerebrovascular disease, enable the discovery of precise biomarkers and provide a novel molecular basis for early detection, disease grading and therapy [96]. Some of the lncRNAs that play important roles in the regulation of neuronal survival, angiogenesis and inflammation may exert significant regulatory effects on neural repair after acute brain injury through intercellular communication mediated by exosomes. The molecular regulatory mechanisms governing neural stem cell proliferation, migration, and differentiation share conserved features with those of other stem cell populations. Exosomal lncRNAs interact with specific miRNAs to regulate the expression of downstream target genes and participate in the formation of various pivotal pathophysiological processes including neuronal apoptosis, blood–brain barrier function, neuroinflammation and vascular remodeling. Brain-derived exosomes, in addition to expressing classic exosomal markers such as CD9, CD63 and CD81, also express neuron-specific proteins such as neuronal nuclear antigen (NeuN), glial fibrillary acidic protein (GFAP) and vascular endothelial growth factor (VEGF) [97]. Exosomes, which are derived from a patient’s peripheral blood, are specifically labeled by these “disease” markers, and the altered gene expression profiles of lncRNAs can be useful in predicting the level of damage to the brain and functional outcomes.

#### 6.1.1. Exosomal lncRNAs Involved in the Occurrence of Ischemic Stroke

##### Downregulated Expression of Exosomal lncRNAs During Ischemic Stroke

In the pathological process of ischemic stroke, the expression of various exosomal lncRNAs is significantly downregulated, and these changes are closely related to neuronal damage, vascular dysfunction, and neuroinflammation. The lncRNA TINCR is significantly downregulated in cerebral ischemia–reperfusion models. Its overexpression relieves neuronal apoptosis and inflammatory damage by sponging miR-125b-5p and derepressing downstream antiapoptotic genes [98]. Conversely, MALAT1, a key regulator of cerebrovascular endothelial function, aggravates microvascular endothelial cell apoptosis, promotes the release of IL-6 and monocyte chemoattractant protein-1 (MCP-1) release, and compromises blood–brain barrier integrity by upregulating the pro-apoptotic protein Bim [99].

The exosomal lncRNA KLF3-AS derived from BMSCs has significant neuroprotective effects on ischemic stroke, and its expression is negatively associated with the degree of brain injury. Cao et al. [73] revealed that exosomal the lncRNA KLF3-AS1 can upregulate the expression of sphingosine kinase 1 (Sphk1) protein and inhibit the activation of the NF-κB signaling pathway, thereby reducing OGD/R-induced apoptosis and inflammatory injury via inhibition of the NF-κB pathway (as described in Section 5.3). In a middle cerebral artery occlusion (MCAO) model, treatment with the exosomal lncRNA KLF3-AS1 markedly decreased the cerebral infarct volume and improved the neurological function score, which was related to the promotion of neuronal cell viability, inhibition of the apoptotic chain, and inhibition of reactive oxygen species (ROS) production [74]. Further studies revealed that the lncRNA KLF3-AS1 upregulates USP22 expression by sponging miR-206. USP22 then reverses cerebral ischemia–reperfusion injury by Sirt1 downregulation, activating the Sirt1-mediated antioxidative stress pathway. This “KLF3-AS1/miR-206/USP22/Sirt1” regulatory axis thus alleviates ischemia-induced apoptosis, inflammation, and oxidative stress [74]. Although these models incompletely recapitulate human cerebrovascular pathology, sample sizes in existing studies are frequently small. CircRNA BBS2 (circBBS2) expression is downregulated in ischemic stroke patients. The administration of UC-MSC-derived circBBS2 significantly alleviates ischemic stroke in rats by sponging miR-494, enhancing SLC7A11 expression, and inhibiting ferroptosis [100].

##### Upregulated Expression of Exosomal lncRNAs During Ischemic Stroke

The results of this study revealed that the levels of lncRNAs were significantly greater in the blood of ischemic stroke patients’ exosomes than in that of normal individuals. Therefore, these lncRNAs could be used as disease markers for ischemic stroke patients or participate in the regulation of related pathological processes. LncRNA H19 is markedly upregulated in peripheral blood exosomes from ischemic stroke patients and is negatively correlated with blood insulin-like growth factor 1 (IGF1) levels. The lncRNA H19 can be transported via exosomes from neurons to astrocytes, potentially through the let-7a/IGF-1 receptor axis to induce the downregulation of IGF-1 [84]. Moreover, the level of the lncRNA MEG8 in the peripheral blood exosomes of ischemic stroke patients is significantly decreased. Additionally, in an OGD model, MEG8 increased the transcription of vascular endothelial growth factor A (VEGFA) through its ability to sponge miR-130a-5p, leading to increased numbers of vessels during angiogenesis and increased numbers of vessels that connect to collateral circulation. The absence of MEG8 impaired the ability of the patient to repair the damaged vascular system after an ischemic event [101].

The highly conserved nuclear lncRNA MALAT1 is markedly upregulated after cerebral ischemia and plays complex roles in neuroinflammation [102,103]. Although MALAT1 is involved in neuroinflammation and this lncRNA has been reported to have anti-inflammatory effects and is correlated negatively with IL-1β [103], its sustained or elevated expression may paradoxically activate the NF-κB pathway and aggravate the inflammatory cascade, suggesting that time- and dose-dependent effects remain inadequately reconciled in the current literature. In ischemic stroke models, the nucleus-localized lncRNA PEG11as is significantly upregulated in ischemic brain tissues. PEG11as may regulate autophagy through the “PEG11as/miR-342-5p/PFN1” axis, inducing protective autophagy and alleviating early-stage cell damage [103,104].

#### 6.1.2. Exosomal lncRNAs and Hemorrhagic Stroke

Hemorrhagic stroke is essentially defined as the rupture of cerebral blood vessels, resulting in intravascular hemorrhage, hematoma and a series of pathological cascade reactions caused by defects in the structure of the blood vessel wall and corotational disturbance [105]. If not treated in time, hemorrhagic stroke may lead to brain herniation and death. Owing to the increasing prevalence of hypertension and the widespread use of anticoagulant drugs, the incidence of hemorrhagic stroke has increased. Hemorrhagic stroke is a leading cause of long-term neurological dysfunction. In addition to its high mortality rate in the acute phase, long-term survivors suffer from cognitive dysfunction and motor dysfunction, severely impacting patient quality of life. Secondary brain injury after hematoma formation is among the main pathological mechanisms of hemorrhagic stroke. The pathological process is very complex, and involves a variety of factors, including the toxic effects of hemoglobin degradation products, oxidative stress, neuroinflammation, and apoptosis.

There are significant differences in the plasma extracellular vesicles lncRNA expression profiles between patients with hemorrhagic stroke and healthy controls at different stages of the disease course. Compared with control patients, patients with hemorrhagic stroke exhibit significant changes in plasma extracellular vesicles. This alteration is similar to that of age- and sex-matched patients, and the lncRNA profile is correlated with the volume of the hematoma and the neurological functional score [85]. Changes in the expression of lncRNAs within extracellular vesicles after hemorrhagic stroke are greatest during the early acute phase (24–72 h after the onset of stroke), and these changes in expression are associated with the body’s inflammatory response and apoptosis. Mechanistically, the lncRNA TM7SF3-AU1 competitively binds miR-702-3p, derepressing SARM1 and activating the downstream apoptotic cascade, thereby exacerbating white matter injury [85]. In contrast, the MSC-derived exosomal lncRNAs GAS5 and MALAT1 exert protective effects by inhibiting apoptosis, reducing inflammation, and promoting angiogenesis in hemorrhagic stroke models [106,107,108,109]. Therefore, the dynamic assessment of plasma extracellular vesicles of patients after hemorrhagic stroke serves as an important tool for determining the severity of the disease and developing an individualized treatment plan for each patient.

### 6.2. Exosomal lncRNAs and Chronic Cerebrovascular Lesions

Chronic cerebrovascular lesions are a series of diseases that are characterized by gradual injury to the structure and function of cerebral blood vessels, in which atherosclerosis is the main pathological basis [109]. The lesion begins with vascular endothelial injury, after which lipid deposition in the vessel wall leads to atherosclerotic plaques, narrowing the lumen and reducing compliance. Eventually, severe complications such as intracranial aneurysm may develop. In the occurrence and development of chronic cerebrovascular lesions, exosomes play dual regulatory roles as important mediators of intercellular communication. In promoting lesion development, exosomes released by damaged vascular endothelial cells, activated macrophages, and foam cells carry pathogenic molecules that participate in the disease process. The transfer of signals related to redox imbalance, mitochondrial dysfunction and metabolic disturbance begins with macrophage-derived foam cell exosomes; these signals are provided to microglia through the miR-101-3p/Nrf2/Slc2a1 signaling pathway, creating an environment that significantly increases neuroinflammatory responses [110]. Moreover, lncRNAs are important for the transfer of these mechanisms. In the context of atherosclerotic lesions, the expression of GAS5 is upregulated compared with that in control samples. GAS5 contributes to the formation of atherosclerotic plaques by inducing macrophage apoptosis and vascular endothelial apoptosis [86]. Moreover, DUXAP8 participates in the pathological progression of intracranial aneurysms by activating inflammatory signaling pathways through the upregulation of CHPF2 expression [111]. However, exosomes are also involved in endogenous protective mechanisms. After endothelial cell autophagy is activated, miR-204-5p can be packaged into multivesicular bodies and secreted via exosomes. This lncRNA alleviates endothelial dysfunction and apoptosis by targeting BCL2 expression and inhibits vascular smooth muscle cell calcification by regulating the expression of RUNX2 and other factors [112].

#### 6.2.1. Exosomal lncRNAs and Atherosclerosis

##### Decreased Expression of Exosomal lncRNAs During Atherosclerosis

Changes in long noncoding RNA expression profiles during the development of atherosclerosis play important roles in disease progression. LncRNA-FA2H-2 expression is significantly downregulated in atherosclerotic lesions, and its low expression exacerbates ox-LDL-induced vascular endothelial inflammation by upregulating VCAM-1, MCP-1, and IL-6 expression [113]. Downregulated expression of the lncRNA MALAT1 is associated with atherosclerotic plaque formation and increased inflammatory cell infiltration [114], whereas deletion of the lncRNA MIR181A1HG alleviates vascular inflammation and atherosclerosis development [115]. This finding further proves the multiple regulatory functions of lncRNAs in chronic cerebrovascular lesions. The potential of exosomes as disease biomarkers is also gradually being revealed in the diagnosis of chronic cerebrovascular lesions. During atherosclerosis, exosomes derived from the endothelium have increased expression levels of brain vascular-specific proteins. These include glucose transporter-1 (GLUT-1), large neutral amino acid transporter-1 (LAT-1), and P-glycoprotein (P-gp), which reflect the degree of endothelial damage, but exosomes derived from platelets do not show such specific changes. These findings suggest that exosomes from different cellular sources may be specific markers for minimally invasive diagnosis [39].

##### Increased Expression of Exosomal lncRNAs During Atherosclerosis

GAS5, an lncRNA, is a key regulator of atherosclerosis as the disease progresses. The expression levels of this lncRNA are upregulated during atherogenic disease states. GAS5 promotes inflammatory responses and lipid metabolism disorders by sponging miR-21, thereby alleviating the repression of proinflammatory cytokines and lipid metabolism-related genes [116]. Fan et al. further demonstrated that GAS5 overexpression inhibits endothelial autophagy by sponging miR-193-5p, thereby increasing lipid deposition and promoting unstable plaque formation [117]. Chen et al. confirmed that GAS5 is highly upregulated in atherosclerotic plaques in both clinical samples and animal models. Knockdown of GAS5 suppressed ox-LDL-induced THP-1 apoptosis, whereas exosomes from GAS5-overexpressing THP-1 cells promoted vascular endothelial apoptosis, indicating that GAS5 contributes to atherosclerosis progression through exosome-mediated intercellular communication [86].

Various lncRNAs play important regulatory roles in the pathological process of atherosclerosis. Upon vascular endothelial injury, the expression of the lncRNA uc003pxg.1 is upregulated and negatively correlated with that of miR-339-5p. This regulatory axis promotes cell proliferation, migration, and angiogenesis, and upregulates the expression of TGF-β1, α-SMA, CD31, collagen I/III, and endoglin, thereby contributing to vascular remodeling [118]. Under atherogenic conditions, exosomal LOC100129516 is highly expressed and inhibits the PPARγ/LXRα/ABCA1 pathway, thereby reducing cholesterol efflux and promoting foam cell formation. Mesenchymal stem cell-derived exosomes delivering LOC100129516-targeting inhibitors may reverse these effects and restore reverse cholesterol transport, suggesting a potential therapeutic strategy [87].

#### 6.2.2. Exosomal lncRNAs and Aneurysms

The development of cerebral aneurysms is a critical complication of chronic cerebrovascular disease, and is closely related to atherosclerosis. When the arterial wall is damaged by atherosclerotic plaques and the media thins due to atrophy, local outward bulging occurs under continuous blood flow impact, resulting in aneurysmal dilation, which typically occurs at the bifurcations of the intracranial circle of Willis. In the pathogenesis of aneurysms, long noncoding RNAs play important regulatory roles. Vascular smooth muscle cell-derived lncRNA HIF1A-AS1 and lncRNA-p21 participate in aneurysm formation by regulating smooth muscle cell proliferation and apoptosis [119,120]. Wang et al. performed transcriptomic analysis of plasma exosomes from intracranial aneurysm patients and identified 1303 differentially expressed lncRNAs, among which ATP1A1-AS1 was the most significantly upregulated. Functional experiments revealed that ATP1A1-AS1 overexpression promotes vascular smooth muscle cell phenotype switching, induces apoptosis, and upregulates MMP-9 expression, thereby contributing to aneurysm formation [81]. DUXAP8 upregulates CHPF2 expression, regulating endothelial inflammatory responses and promoting aneurysm expansion. Clinically, DUXAP8 and CHPF2 levels positively correlate with aneurysm size (AUC = 0.943) [111].

In abdominal aortic aneurysm research, M1 macrophage-derived PVT1 competitively binds to miR-186-5p, derepressing HMGB1 and activating the NLRP3 inflammasome. This triggers vascular smooth muscle cell pyroptosis and the release of TNF-α and IL-6, accelerating abdominal aortic aneurysm progression [88]. The lncRNA H19 can act as a competing endogenous RNA to enhance aortic inflammatory responses, thus promoting the formation of abdominal aortic aneurysms [121]. During the formation of thoracic aortic aneurysms, the lncRNA HOXA-AS2 promotes vascular smooth muscle cell proliferation via the miR-520d-3p/IGF2BP3 axis [122], whereas lncRNA-p21 regulates cell proliferation and apoptosis through activation of the TGF-β1 signaling pathway [120].

#### 6.2.3. Exosomal lncRNAs Provide a New Strategy for Identifying Vascular Dementia

Vascular dementia (VaD) is a major cognitive disorder resulting from chronic cerebrovascular pathology and is characterized by impairments in executive function, memory, and language ability. In recent years, exosome-based diagnostics have attracted increasing interest in the context of neurodegenerative and vascular cognitive disorders. Plasma extracellular vesicles are particularly advantageous because of their easy accessibility and ability to reflect pathophysiological changes in the brain.

**Figure 4:**
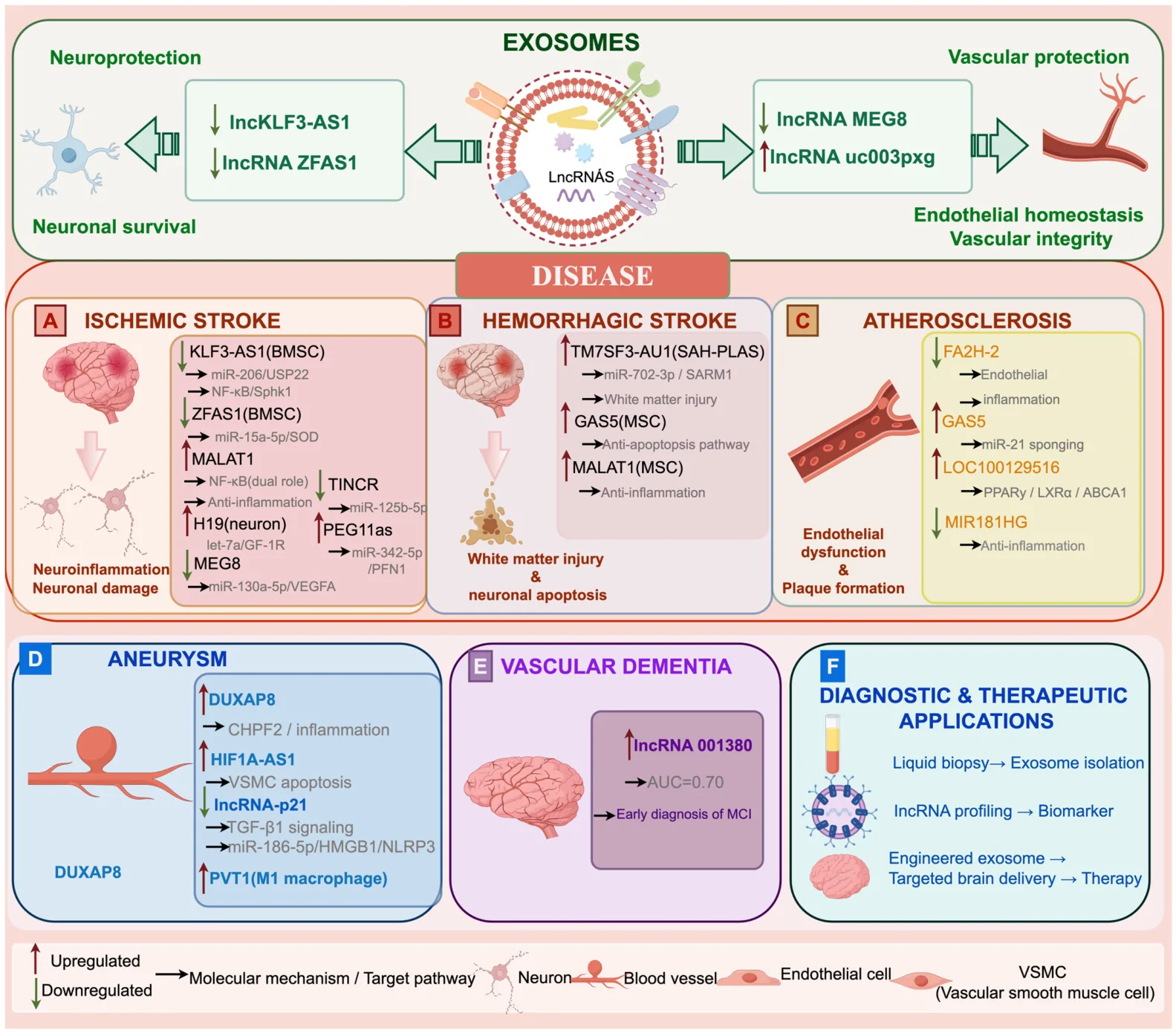
Disease-specific roles and translational applications of exosomal lncRNAs in cerebrovascular diseases. The figure illustrates the principal exosomal lncRNAs involved in four major cerebrovascular disease categories and their proposed translational applications. (**A**) Ischemic Stroke: Key exosomal lncRNAs including KLF3-AS1 (BMSC-derived), ZFAS1 (BMSC-derived), MALAT1, H19 (neuron-derived), and MEG8, along with their molecular targets (miR-206/USP22, miR-15a-5p/SOD, NF-κB/Sphk1, let-7a/IGF-1R and miR-130a-5p/VEGFA) and functional outcomes in neuroinflammation and neuronal damage modulation. (**B**) Hemorrhagic Stroke: TM7SF3-AU1 (SAH patient plasma-derived from patients with SAH) mediates white matter injury and neuronal apoptosis via the miR-702-3p/SARM1 pathway, while MSC-derived GAS5 and MALAT1 exert protective effects through antiapoptotic and anti-inflammatory mechanisms. (**C**) Atherosclerosis: exosomal lncRNAs FA2H-2, GAS5, LOC100129516, and MIR181A1HG participate in endothelial dysfunction and plaque formation through distinct molecular mechanisms including miR-21 sponging and PPARγ/LXRα/ABCA1 pathway regulation. (**D**) Aneurysm: DUXAP8 promotes inflammation via CHPF2 upregulation; HIF1A-AS1 induces VSMC apoptosis; lncRNA-p21 modulates TGF-β1 signaling; and PVT1 (M1 macrophage-derived) activates the NLRP3 inflammasome through miR-186-5p/HMGB1 to drive pyroptosis. (**E**) Vascular Dementia: Plasma LINC001380 serves as a potential diagnostic biomarker for mild cognitive impairment (AUC = 0.70). (**F**) Diagnostic and Therapeutic Applications: Proposed translational workflow encompassing liquid biopsy-based exosome isolation, lncRNA profiling for biomarker discovery, and engineered exosome-mediated targeted brain delivery for therapy. Upward arrows (↑) denote upregulated expression; downward arrows (↓) denote downregulated expression in disease states. The cellular sources of the exosomes are annotated in parentheses. BMSC, bone marrow mesenchymal stem cell; MSC, mesenchymal stem cell; SAH-PLAS, plasma from subarachnoid hemorrhage patients; VSMC, vascular smooth muscle cell; MCI, mild cognitive impairment; AUC, area under the curve.

Xie et al. verified that BMSC-derived exosomal lncRNA KLF3-AS1 alleviated cerebral ischemia–reperfusion injury by reducing ROS accumulation, inhibiting neuronal apoptosis and inflammation, and upregulating Sirt1 expression [74]. Furthermore, plasma LINC001380 has been identified as a potential diagnostic marker for mild cognitive impairment, with its expression level significantly correlated with the severity of cognitive deficit (AUC = 0.70) [123]. These findings suggest that exosomal lncRNAs may serve as valuable tools for the early detection and monitoring of vascular cognitive impairment, although further validation in larger cohorts is needed. A comprehensive overview of the disease-specific roles, molecular mechanisms, and translational applications of exosomal lncRNAs in cerebrovascular diseases is presented in Figure 4.

## 7. Challenges and Future Perspectives

### 7.1. Challenges Restricting the Clinical Transformation of Exosomal lncRNAs

Exosomes possess promising therapeutic potential but encounter multiple bottlenecks hindering clinical translation. Primarily, exosome heterogeneity derived from parental cell types, individual vesicle variations and culture conditions leads to inconsistent internal cargos, variable therapeutic effects and poor batch repeatability, greatly obstructing unified clinical dose formulation [124,125]. Second, common purification technologies such as ultracentrifugation, size-exclusion chromatography and precipitation all have defects, and there is no globally unified GMP standard purification workflow. Divergent quantification standards using particle number or total protein make clinical trial data hard to compare across research teams [126,127]. Third, natural exosomes are rapidly cleared in vivo and have a short circulation half-life and unsatisfactory target accumulation. Current drug loading methods feature low encapsulation efficiency. Long-term safety hazards including potential immune reactions and abnormal cell proliferation lack long-term follow-up evidence, but unclear dose–effect relationships impede standardized medication plans [128,129]. Finally, global regulatory standards are inconsistent for exosome products. Most medical institutions lack dedicated detection devices and complete cold chain systems. High GMP manufacturing costs, inadequate large-sample phase III clinical evidence and insufficient clinician and patient understanding collectively block large-scale clinical promotion [130].

### 7.2. Advances and Future Perspectives

Compared with standard cell therapy, exosomes are nonreplicative and highly controllable, and they exhibit low immunogenicity, with therapeutic efficacy validated in numerous animal models of chronic cerebrovascular disorders. Traditional bulk exosome analysis cannot fully resolve vesicle heterogeneity, restricting the accurate identification of pathological molecular alterations. In comparison, single-cell profiling achieves high-resolution mapping of exosomal molecular signatures, enabling precise mechanistic exploration and specific biomarker screening [131,132]. Furthermore, artificial intelligence (AI) and machine learning (ML) approaches have emerged as powerful tools for accelerating exosomal biomarker discovery. By integrating multiomics datasets, ML algorithms can identify exosomal lncRNA expression signatures with higher diagnostic sensitivity and specificity than conventional single-marker strategies can, facilitating stroke subtype classification and prognostic prediction [133,134]. Exosomal lncRNAs participate broadly in the pathophysiological processes of cerebrovascular diseases through diverse molecular mechanisms, including miRNA sponging, signaling pathway protein regulation, and alternative splicing (as detailed in Section 5 and Section 6). These mechanisms collectively modulate key pathological events such as apoptosis, inflammation, and oxidative stress, thereby influencing disease onset and progression. Notably, CRISPR/Cas9-based genome editing has opened new avenues for the precise modulation of ncRNA targets in cerebrovascular diseases. For example, CRISPR-Cas9-mediated knockout of the stroke-responsive lncRNA FosDT significantly reduced infarct size and improved motor function recovery in rodent ischemic stroke models, indicating that lncRNAs are actionable therapeutic targets [135,136]. Moreover, CRISPR-Display technology enables the targeted recruitment of functional lncRNA domains to specific genomic loci without permanent genomic alteration, providing an innovative platform for dissecting lncRNA regulatory mechanisms in cerebral ischemia [137].

Moreover, engineered exosome systems effectively shield encapsulated lncRNAs from in vivo degradation and enable targeted brain delivery [138,139]. Compared with cell therapies, exosome-based interventions exhibit superior controllability, low immunogenicity, and a favorable safety profile, with consistent efficacy verified in diverse preclinical models. Nevertheless, multiple hurdles still hinder clinical translation. Existing studies remain largely preclinical, with incompletely defined molecular mechanisms, insufficient metabolic and long-term safety data, and a lack of standardized manufacturing, quality control, and large-scale clinical validation. Looking ahead, the convergence of AI-driven biomarker panels, CRISPR-mediated lncRNA functional validation, and exosome-based targeted delivery holds the potential to advance personalized medicine for cerebrovascular diseases—enabling individualized risk stratification through exosomal lncRNA liquid biopsy signatures, patient-specific therapeutic lncRNA selection, and precision-guided brain-targeted interventions [140,141]. The future technical optimization of exosome purification and bioengineering, combined with unified operational specifications and multicenter clinical trials, may accelerate the clinical implementation of exosomal lncRNAs in early diagnosis, dynamic surveillance, and targeted therapy, advancing precision medicine for cerebrovascular diseases.

## Data Availability

No new data were created or analyzed in this study. Data sharing is not applicable to this article.

## Abbreviations

The following abbreviations are used in this manuscript:

AAA	Abdominal Aortic Aneurysm
AP2	adaptor protein complex 2
ATP	adenosine triphosphate
Aβ	amyloid-beta
BAR	Bin Amphiphilic Rvs
BMSC	Bone Marrow Mesenchymal Stem Cell
Cav1	Caveolin-1
ceRNAs	competitive endogenous RNAs
CircRNA	circular RNA
CIRI	Cerebral ischemia–reperfusion injury
CSF	cerebrospinal fluid
ESCRT	endosomal sorting complex required for transport
EVs	extracellular vesicles/exosomes
GPCRs	G protein-coupled receptors
GPx	glutathione peroxidase
Hsp70	Heat shock protein 70
iNOS	inducible nitric oxide synthase
LPS	lipopolysaccharide
lncRNA	Long noncoding RNA
MCAO/R	middle cerebral artery occlusion/reperfusion
MDA	malondialdehyde
MHC	major histocompatibility complex
MSC	Mesenchymal Stem Cell
NF-κB	nuclear factor kappa-B
NV	neuron-derived exosomes
OGD/R	oxygen glucose deprivation/reoxygenation
PD	Parkinson’s disease
PI3K/Akt	Phosphoinositide 3-kinase/Protein kinase B
PV	platelet-derived exosomes
ROS	reactive oxygen species
RNS	reactive nitrogen species
SOD	superoxide dismutase
TLR4	Toll-like receptor 4
V-ATPase	vacuolar-type H^+^-ATPase
VSMC	Vascular Smooth Muscle Cell
